# Enzymatic Biotransformation of Gypenoside XLIX into Gylongiposide I and Their Antiviral Roles against Enterovirus 71 In Vitro

**DOI:** 10.3390/molecules27134094

**Published:** 2022-06-25

**Authors:** Huanxi Zhao, Wenbo Jiao, Yang Xiu, Kailu Zhou, Peng Zhong, Nan Wang, Shanshan Yu

**Affiliations:** 1 Jilin Ginseng Academy, Changchun University of Chinese Medicine, Changchun 130117, China; zhaohx@ccucm.edu.cn (H.Z.); xiuyang@ccucm.edu.cn (Y.X.); zhoukl@ccucm.edu.cn (K.Z.); zhongpeng@ccucm.edu.cn (P.Z.); wangnan@ccucm.edu.cn (N.W.); 2 Department of Clinical Laboratory, Affiliated Hospital of Changchun University of Chinese Medicine, Changchun 130117, China; lvxy@ccucm.edu.cn

**Keywords:** *Gynostemma pentaphyllum* (Thunb.) Makino, biotransformation, gypenoside XLIX, gylongiposide I, Enterovirus 71

## Abstract

Biotransformation of specific saponins in the valuable medical plants to increase their bioavailability and pharmaceutical activities has attracted more and more attention. A gene encoding a thermophilic glycoside hydrolase from *Fervidobaterium pennivorans* DSM9078 was cloned and expressed in *Escherichia coli*. The purified recombinant enzyme, exhibiting endoglucanase cellulase activity, was used to transform gypenoside XLIX into gylongiposide I via highly selective and efficient hydrolysis of the glucose moiety linked to the C21 position in gypenoside XLIX. Under the optimal reaction conditions for large scale production of gylongiposide I, 35 g gypenoside XLIX was transformed by using 20 g crude enzyme at pH 6.0 and 80 °C for 4 h with a molar yield of 100%. Finally, 11.51 g of gylongiposide I was purified using a silica gel column with 91.84% chromatographic purity. Furthermore, inhibitory activities of gypenoside XLIX and gylongiposide I against Enterovirus 71 (EV71) were investigated. Importantly, the EC_50_ of gypenoside XLIX and gylongiposide I calculated from viral titers in supernatants was 3.53 μM and 1.53 μM, respectively. Moreover, the transformed product gylongiposide I has better anti-EV71 activity than the glycosylated precursor. In conclusion, this enzymatic method would be useful in the large-scale production of gylongiposide I, which would be a novel potent anti-EV71 candidate.

## 1. Introduction

*Gynostemma pentaphyllum* (Thunb.) Makino (GpM), named “south ginseng”, is a precious medicinal herb which has been widely used all over the world in the past decade. It is easier to obtain than medicinal *Panax ginseng*, whose cultivation is highly specialized and takes 5–6 years to mature [1]. Hence, *G. pentaphyllum* has attracted continuous research interest.

Gypenosides are the principal components responsible for the diverse and significant effects of *G. pentaphyllum*. The pharmacological activities include anti-cancer [2], anti-inflammatory [3], anti-viral [4], anti-atherogenic [5], antioxidant [6], anti-hyperglycemia [7] and neuroprotective activities [8]. Until now, more than 300 gypenosides have been isolated and structurally elucidated from *G. pentaphyllum* [9]. They display dammarane-style structure of tetracyclic triterpenes, which consist of sapogenins, sugars, uronic acid and other organic acids. It has been demonstrated that structural differences such as linked positions, numbers and types of sugar moieties in gypenosides contribute to their different pharmacological activities. In addition, gypenosides with less glucosyl are proven to be more pharmaceutically active. For instance, Zheng et al. reported that gypenoside TN-1 deglycosylated from gypenoside XLVI by using naringinase showed significantly higher inhibitory effect on SMMC7721 and Bel7402 hepatoma cells than the glycosylated precursor [10]. Cui et al. also demonstrated that gypenoside LXXV, which is one of the deglycosylated shapes of gypenoside XVII, significantly reduced cell viability and displayed an enhanced anti-cancer effect compared to gypenoside XVII [11]. Hence, transformation of glycosylated gypenosides through hydrolysis of the sugar moieties in gypenosides provides a new way to obtain more pharmacologically active components.

Various transformation approaches such as physiochemical methods including heating, acid treatment and alkali treatment, and bioconversion methods using microorganisms and enzymes have been attempted [12]. Among these methods, the enzymatic conversion is the most promising method because of its high substrate specificity and stability, low levels of by-products, and high production yields. To minimize processing time and production cost, recombinant enzymes obtained from *E. coli* strains are commonly applied for the gypenosides conversion. For instance, the recombinant β-glucosidase from *Lactobacillus brevis* was identified to transform gypenoside XVII into Compound K (CK) [13]. Compound K, one of the deglycosylated saponin, could be generated by the hydrolysis of sugar moieties from the gypenoside XVII. Higher productivity was achieved when recombinant thermophilic enzymes were used to transform gypenosides. The recombinant β-glucosidase from *Thermus thermophilus* converted the gypenoside XVII to F2 with a molar yield of 100% [14]. However, the number of gypenoside-transforming glycosidases is still limited. Additionally, the thermostability, transformation activity and specificity of most enzymes still do not meet the industrial demands. It is therefore meaningful to explore novel gypenoside-transforming glycosidases with good thermostability, high catalytic efficiency and specificity.

Enteroviruses 71 (EV71), single-stranded, neurotrophic RNA virus, causes countless outbreaks of hand, foot, and mouth disease (HFMD) in hundreds of thousands of young children. It possesses strong neurotropism, leading to severe illness or sudden death. So far, no antiviral agent against EV71 has yet been licensed and approved worldwide. Therefore, there is an urgent need to develop antiviral drugs against EV71. Extracts of Chinese herbs have been used against newly emerging viruses such as SARS-CoV [15] and COVID-19 [16]. Gypenosides from *G. pentaphyllum* also possess antiviral effects [17]. Yang et al. demonstrated that gypenoside inhibited bovine viral diarrhea virus replication by interfering with viral attachment and internalization and by activating apoptosis of infected cells [4]. Crude extracts of *G. pentaphyllum* were also proven to have antiviral activity against highly pathogenic H5N1 avian influenza virus [18]. However, there is still a lack of research on the antiviral activities of gypenosides against EV71.

Gypenoside XLIX is one of the major active gypenosides in *G. pentaphyllum*. Its content is highest (6–20%) in total gypenosides extract. Increasing evidence shows that gypenoside XLIX displays various pharmacological effects such as inhibiting tumor growth, protecting cardiovascular and cerebrovascular system, reducing blood sugar, regulating blood lipid and kidney-protective activity [19,20,21,22]. Moreover, it shows superior anti-inflammatory effects [23]. Gypenoside XLIX is a dammarane-style structure of tetracyclic triterpenes, which has one glucose located at the C21 position and a trisaccharide moiety consisting of one arabinose, one rhamnose and one xylose connected to the C3 position of the aglycone. Considering that the hydrolysis of sugar moieties linked to gypenoside XLIX may improve its pharmacological activity, the purpose of this study is to establish an effectively enzymatic method to convert gypenoside XLIX into deglycosylated saponins, so as to lay a foundation for the pharmacological activity research and application development.

In our research, large-scale biotransformation of gypenoside XLIX into gylongiposide I by using enzymatic method was studied. Moreover, the antiviral effects of gypenoside XLIX and its deglycosylated product gylongiposide I against EV71 in vitro were investigated. The results of the antiviral activities of gypenoside XLIX and gylongiposide I would shed light on alternative therapeutic sources for treatment of EV71 infection in the future.

## 2. Results

### 2.1. Expression and Purification of the Cellulase from Fervidobaterium pennivorans DSM9078

The putative cellulase gene consisted of 969 bp encoding 323 amino acids with a theoretical molecular mass of 37.89 kDa and a theoretical pI value of 5.43. The gene was cloned and expressed in Escherichia coli under the control of the IPTG-inducible promoter T7. After being induced under 16 °C for 12 h with 1 mM IPTG, the recombinant enzyme was solubly overexpressed in *E. coli* cells and was purified by His-trap affinity chromatography. The purified enzyme displayed high activity towards CMC (297 U/mg), but undetectable activity on Avicel and p-nitrophenyl β-d-glucopyranoside (pNPG), indicating that it was an endoglucanase cellulase.

### 2.2. Characterization of the Recombinant Enzyme for Gypenoside XLIX Transformation

**Figure 1:**
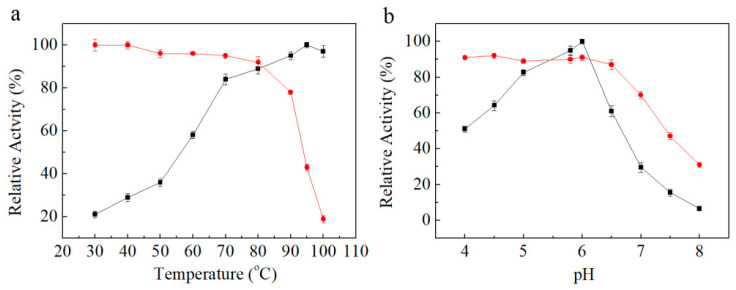
(**a**) Effect of temperature on enzyme activity (■) and stability (●). (**b**) Effect of pH on enzyme activity (■) and stability (●). The activities were determined by using gypenoside XLIX as substrate via HPLC analysis.

The effects of temperature and pH on activity and stability of the recombinant enzyme for gypenoside XLIX transformation were determined via HPLC analysis. As seen in Figure 1a,b, the maximum activity was observed at 95 °C and pH 6.0. After incubation the enzyme at different pHs from 4.0 to 8.0 (at 80 °C) for 1 h, more than 90% of the biotransformation activity remained at acid pHs, while 70% of the activity remained at pH 7.0, indicating that the enzyme was more stable at acid pHs (Figure 1b). Furthermore, the enzyme was very stable at temperatures range from 30−80 °C. After incubation for 12 h, thermostability tended to decrease at or above 90 °C (Figure 1a). Though the recombinant enzyme had highest temperature at 95 °C, biotransformation was occurred at 80 °C for extension of stable transformation activity.

### 2.3. Structural Analysis of the Biotransformed Product of Gypenoside XLIX

**Figure 2:**
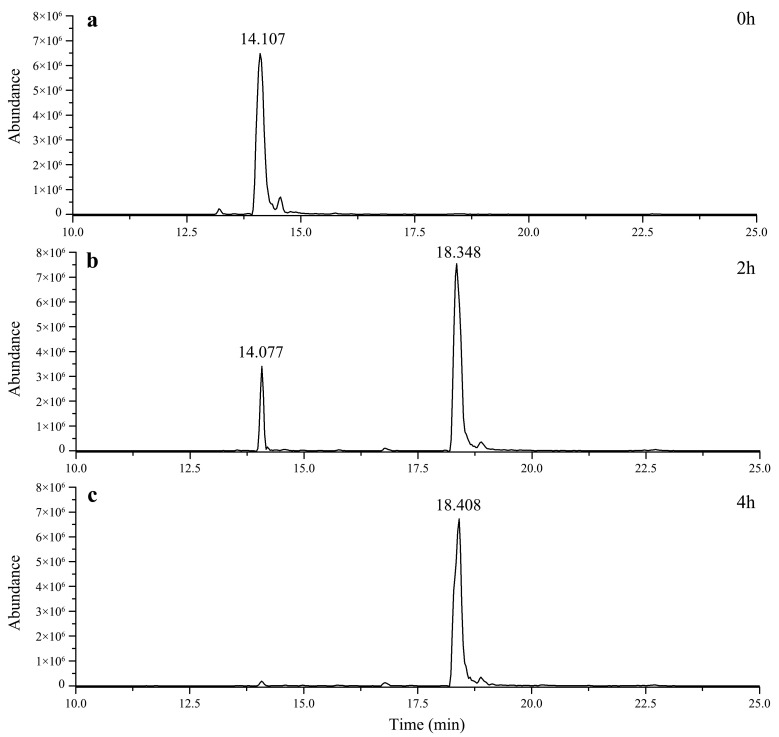
Total ion chromatograms for the enzymatic transformation of gypenoside XLIX by using the recombinant enzyme for 0 h (**a**), 2 h (**b**) and 4 h (**c**).

The biotransformation process of gypenoside XLIX by using the recombinant enzyme was studied via HPLC-MS. As indicated in Figure 2a, gypenoside XLIX had a retention time of 14.107 min. After transformation for 2 h, its peak area decreased dramatically, and one new peak was observed at the retention time of 18.348 min (Figure 2b). Gypenoside XLIX was rather converted after 4 h. Additionally, no more transformed product was observed with the extension of reaction time (Figure 2c).

**Figure 3:**
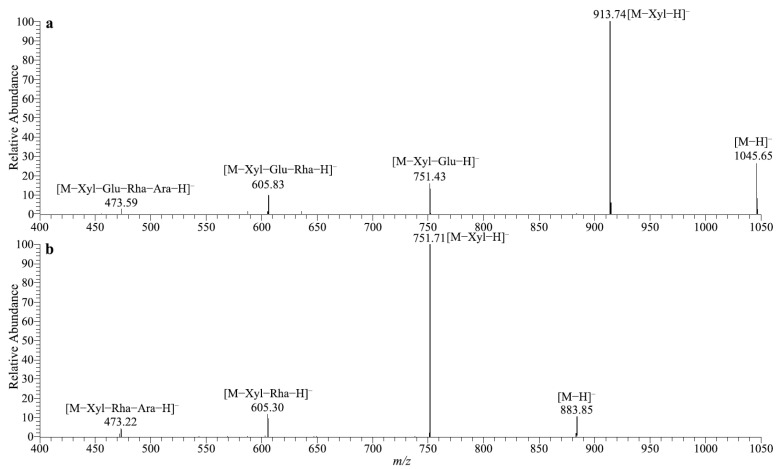
MS/MS spectra of gypenoside XLIX (**a**) and its transformed product (**b**).

The chemical structures of gypenoside XLIX and the transformed product were tentatively investigated by tandem mass spectrometry. The molecular weight of gypenoside XLIX was calculated to be 1046 based on its [M−H]^−^ ion at *m/z* 1045 and [M+HCOO]^−^ ion at *m/z* 1091, corresponding to a molecular formula of C_46_H_76_O_16_. Similarly, the molecular weight of the transformed product was determined to be 884. The mass difference between 1046 and 884 was 162, which corresponded to a glucose moiety. As shown in the tandem MS spectrum of the [M−H]^−^ ion (Figure 3a), four product ions were clearly observed at *m/z* 913, 751, 605 and 473. The neutral losses between the five adjacent ions were 132 u, 162 u, 146 u and 132 u, corresponding to the xylose (Xyl), glucose (Glu), rhamnose (Rha), and arabinose (Ara). In addition, the ion at *m/z* 473 was the characteristic deprotonated aglycone ion of gypenoside XLIX. The tandem MS spectrum of the biotransformed product was similar to that of gypenoside XLIX (Figure 3b). The same product ions at *m/z* 751, 605 and 473 indicated that the Xyl, Rha and Ara residues were reserved during the biotransformation process. Hence, the trisaccharide moiety at the C3 position of gypenoside XLIX was not hydrolyzed. Consequently, it was proposed that gypenoside XLIX was transformed by the recombinant enzyme through deglycosylation of the Glu residue at the C21 position of the aglycone.

**Figure 4:**
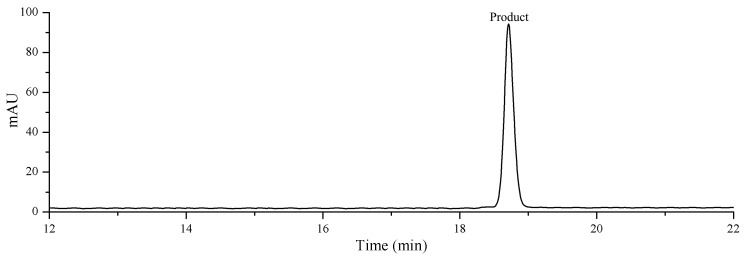
HPLC analysis of the purified gylongiposide I using Prep-HPLC.

In order to further confirm the structure, ^13^C NMR analysis of the biotransformed product was performed. The product was isolated and purified via prep-HPLC. The purity of the collected product was calculated to be 96.82% by the normalization of the peak areas using HPLC (Figure 4), which could meet the requirement of NMR analysis. The ^13^C NMR spectrum showed the presence of one carbonyl group (δC 206.6), two olefinic carbons (δC 124.6, 130.6) and three sugars (anomeric C at δC 100.6, 103.7, 103.8). The ^13^C NMR data was presented in Table S1 (see Supplementary Materials), which were the same as those reported in the previous article [24]. Therefore, the biotransformed product of gypenoside XLIX was elucidated as 3-O-{[α-L-rhamnopyranosyl-(1 → 2)]-[β-D-xylopyranosyl-(1 → 3)]}-α-L-arabinopyranoside-3, 20, 21-trihydroxy, 19-aldehydedammar-24-ene, namely gylongiposide I.

The structural difference between gylongiposide I and gypenoside XLIX lies only in the number of glucose residues at the C21 position of the aglycone. Gylongiposide I was generated by hydrolysis of one glucose residue at C21 position of gypenoside XLIX. Since no other enzymes were introduced into the reaction system, we believe that the conversion of gypenoside XLIX to gylongiposide I is a glucoside hydrolysis reaction catalyzed by recombinant glycoside hydrolase from *Fervidobaterium pennivorans* DSM9078.

**Figure 5:**
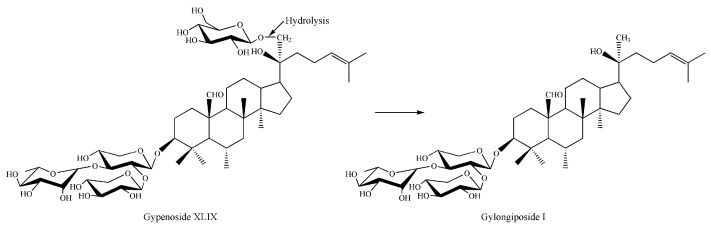
The biotransformation pathway of gypenoside XLIX by using the recombinant enzyme.

The biotransformation pathway was illustrated in Figure 5, gypenoside XLIX was transformed into gylongiposide I by using the recombinant enzyme via the hydrolysis of the glucose moiety at the C21 position of the aglycone.

### 2.4. Large-Scale Production of Gylongiposide I by Using the Recombinant Enzyme

To facilitate scaling up of the production, the recombinant *E. coli* BL21 cells were obtained by high-cell-density fermentation. Cell pellets were resuspended and disrupted by sonication. The supernatant was collected by centrifugation and treated as crude enzyme. As crude enzyme had similar hydrolysis activity to purified enzyme and was more easily obtained, the crude enzyme was used for the large-scale biotransformation.

**Figure 6:**
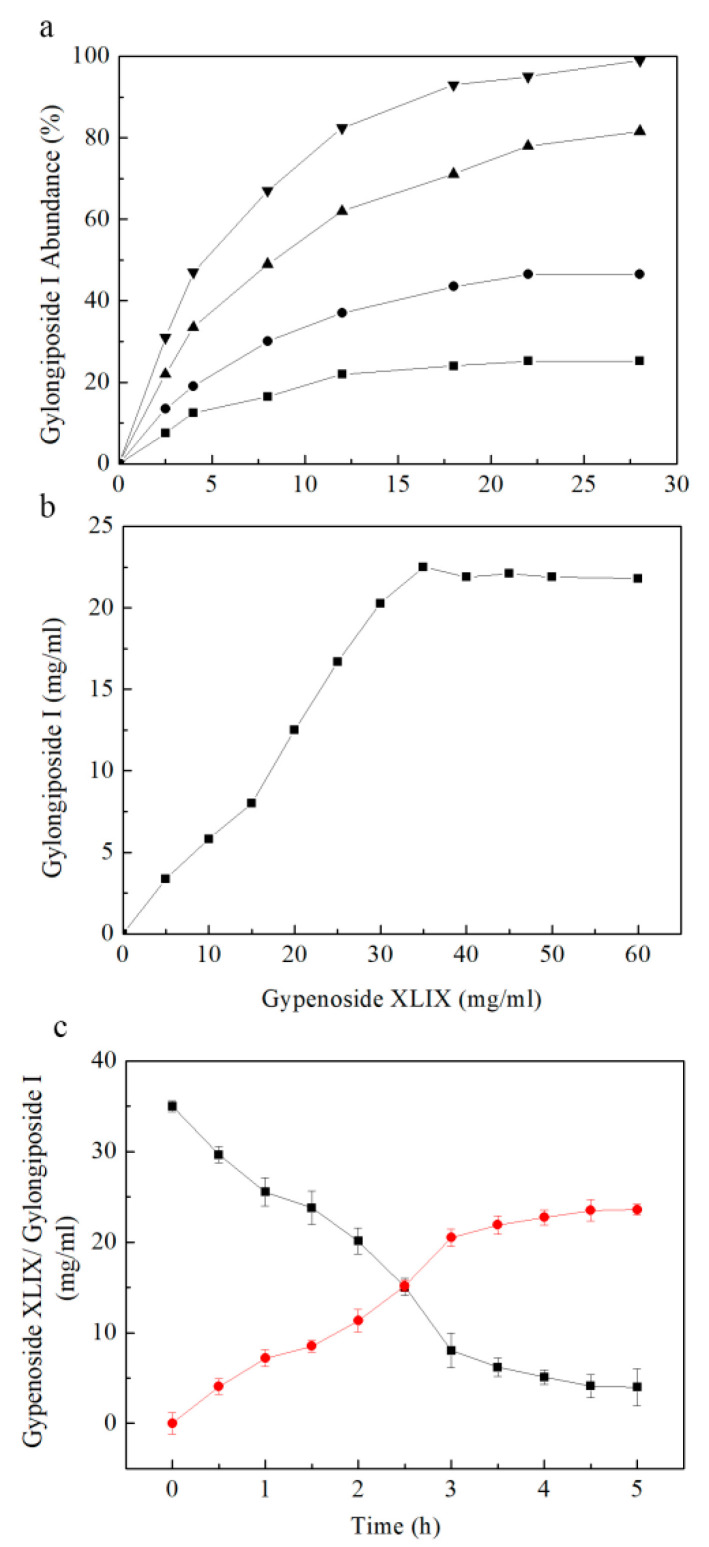
(**a**) Effect of the concentrations of crude enzyme including 2.5 mg/mL enzyme (■), 5 mg/mL enzyme (●), 10 mg/mL enzyme (▲) and 20 mg/mL enzyme (▼) on gylongiposide I production by using the recombinant enzyme. (**b**) Effect of the concentrations of gypenoside XLIX on gylongiposide I production by using the recombinant enzyme. (**c**) Effect of the transformation time on gylongiposide I production (●) from gypenoside XLIX (■) by using the recombinant enzyme.

In order to reduce production costs, the effect of crude enzyme concentration on gylongiposide I production was investigated with 25 mg/mL gypenoside XLIX as the substrate by varying the enzyme concentration from 2.5–20 mg/mL. Gylongiposide I production increased with increasing the enzyme concentration. The conversion of gypenoside XLIX reached 100% using 20 mg/mL enzyme within 3 h (Figure 6a), indicating that the enzyme concentration was optimal at 20 mg/mL.

In consideration of decreasing the reactor volume, the effect of substrate concentration on gylongiposide I production was assessed by varying the concentration of gypenoside XLIX from 0 to 60 mg/mL and reacting with 20 mg/mL enzyme. The gylongiposide I production increased with increasing gypenoside XLIX concentration and reached a plateau above 35 mg/mL (Figure 6b). Thus, the optimal substrate concentration was determined to be 35 mg/mL.

In order to determine the most appropriate reaction time, the time course of the biotransformation of gypenoside XLIX to gylongiposide I was monitored via HPLC analysis. As seen in Figure 6c, the production of gylongiposide I reached its highest level after 4 h biotransformation, and the gypenoside XLIX was transformed completely.

Hence, the scaled-up biotransformation was performed in a 5.0 L glass bottle (2.0 L working volume) under optimal conditions (shaking 200 rpm for 4 h at pH 6.0 and 80 °C). The crude recombinant enzyme (20 mg/mL) was reacted with an equal volume of gypenoside XLIX (35 mg/mL). Under the optimal reaction conditions, gypenoside XLIX was transformed to gylongiposide I with a molar yield of 100%.

### 2.5. Separation and Purification of Transformed Gylongiposide I

In order to obtain gylongiposide I of high purity, the enzymes, salts, and free sugars in the reaction mixture were removed by HP-20 macroporous resin. A total of 23.76 g of crude gylongiposide I was obtained by HP-20 macroporous resin and further purified through a silica gel column. Then, 11.51 g of gylongiposide I was finally achieved with a purity of 91.84%, as determined by HPLC analysis.

### 2.6. Inhibitory Effect of Gypenoside XLIX and Gylongiposide I on EV71 Replication

**Figure 7:**
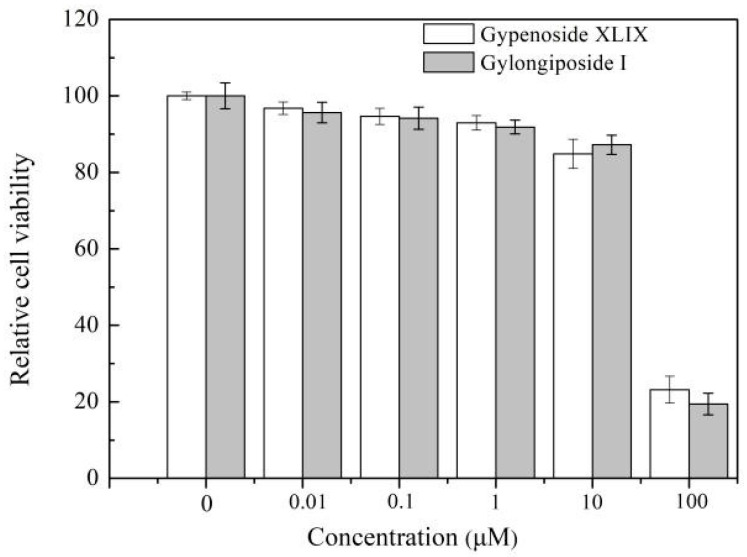
Dose-dependent cytotoxicity profiles of gypenoside XLIX and gylongiposide I were detected by a CCK8 kit in RD cells at 48 h. Inhibitory effect of gypenoside XLIX on EV71 replication in the 0.01 to 10 µM dose range.

**Figure 8:**
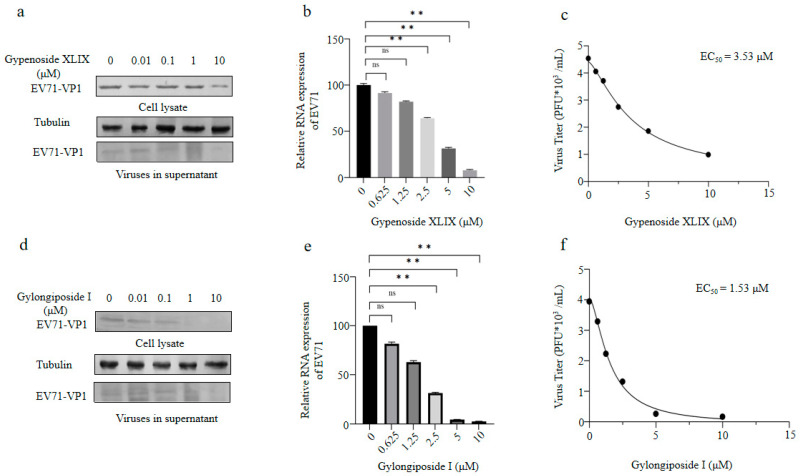
Anti-EV71 activity of gypenoside XLIX and gylongiposide I. (**a**) The inhibitory effect of gypenoside XLIX on EV71 replication in the 0–10 µM dose range. RD cells were infected with EV71 at an MOI of 2 for 4 h, washed twice with DMEM and supplemented with DMEM containing the indicated concentration of gypenoside XLIX for another 48 h. The cells were harvested for Western blot (WB) analysis, and tubulin was used as a loading control. The supernatant from the infected RD was centrifuged and then loaded for WB analysis. (**b**) Cellular EV71 RNA levels in Figure 7b were detected by RT-qPCR. GAPDH was used as a control. The EV71 RNA level without gypenoside XLIX treatment was set as 100%. Significant differences are indicated in figures as follows: ** *p* ≤ 0.01, ns stands for not significant. (**c**) Viral titers in the supernatants from the experiment shown in Figure 7b were determined by the plaque assay. The EC_50_ of gypenoside XLIX was calculated by GraphPad Prism7. (**d**) The inhibitory effect of gylongiposide I on EV71 replication in the 0–10 µM dose range. RD cells were infected with EV71 at an MOI of 2 for 4 h, washed twice with DMEM and supplemented with DMEM containing the indicated concentration of gylongiposide I for another 48 h. The cells and supernatants were harvested for WB analysis. (**e**) Cellular EV71 RNA levels in Figure 7e were detected by RT-qPCR. GAPDH was used as a control. The EV71 RNA level without gylongiposide I treatment was set as 100%. Significant differences are indicated in figures as follows: ** *p* ≤ 0.01, ns stands for not significant. (**f**) Viral titers in the supernatants from the experiment shown in Figure 7e were determined by the plaque assay. The EC_50_ of gylongiposide I was calculated by GraphPad Prism7. The results shown are the means with SDs from two independent experiments.

Previous studies have shown that gypenoside XLIX has potential anti-bacterial or anti-inflammatory activities. However, its antiviral function remains unknown. Enterovirus 71 is an important pathogen causing hand, foot and mouth disease. Here, the anti-EV71 effects of gypenoside XLIX and its biotransformed product gylongiposide I was investigated. Cytotoxicity test showed that the two compounds had no obvious toxicity to RD cells, when cells were treated with them below 10 μM (Figure 7). Meanwhile, 10 μM gypenoside XLIX and 1 μM gylongiposide I clearly decreased viral protein VP1 expression in cell lysate and cultural supernatant, when RD cells were incubated with 0.01−10 μM of gypenoside XLIX or gylongiposide I prior to EV71 infection (Figure 8a,d). The antiviral effects were also validated by measuring the intracellular viral genomic RNA copies and the extracellular viral titer. As shown in Figure 8b,e, gypenoside XLIX and gylongiposide I suppressed EV71 mRNA transcripts in RD cells both in a dose dependent manner. Importantly, the EC_50_ of gypenoside XLIX and gylongiposide I calculated from viral titers in supernatants was 3.53 μM and 1.53 μM, respectively (Figure 8c,f). Our data suggested that gypenoside XLIX and gylongiposide I exhibited anti-EV71 effect in vitro, and the transformed product gylongiposide I had better anti-EV71 activity than the precursor gypenoside XLIX.

## 3. Discussion

Biotransformation of specific saponins in the valuable medical plants to increase their production and bioactivity have attracted more and more attention. Several studies have been published on the bioconversion of gypenosides by using recombinant enzymes. For example, recombinant β-glucosidase from *Microbacterium* sp. Gsoil 167 efficiently hydrolyzed gypenoside XVII into gypenoside LXXV [11]. The recombinant β-glucosidase from *Lactobacillus brevis* transformed gypenoside XVII into compound K (CK). Until now, the number of recombinant gypenoside-transforming enzymes is still limited. Additionally, the biotransformation reactions only occurred on gypenoside XVII or gypenoside LXXV. The activity, specificity, and thermostability of most enzymes still do not meet the industrial demands.

The cellulase gene was cloned from *F. pennivorans* DSM9078, which is a thermophilic bacterium. The recombinant enzyme was active and stable under acidic conditions. It exhibited optimal activity at pH 6.0, which is similar to glycosidases from thermophilic origins such as β-glucosidases from *Sulfolobus solfataricus* [25], *Pyrococcus furiosus* DSMZ 3638 [26] and *Thermotoga thermarum* DSM 5069T [27]. The optimal temperature of the enzyme was about 95 °C. It also displayed high thermostability. Thermostable enzymes serve as ideal catalysts for biotransformation application because high temperatures improve the ginsenosides solubility, enhance the substrate conversion and reduce the need for expensive cooling process. For instance, the thermophilic β-glucosidase from *Thermus thermophilus* converted the gypenoside XVII to F2 with a molar yield of 100% [14]. Similarly, in our research, 35 g gypenoside XLIX was transformed by using 20 g crude enzyme at pH 6.0 and 80 °C within 4 h with a molar yield of 100%.

**Figure 9:**
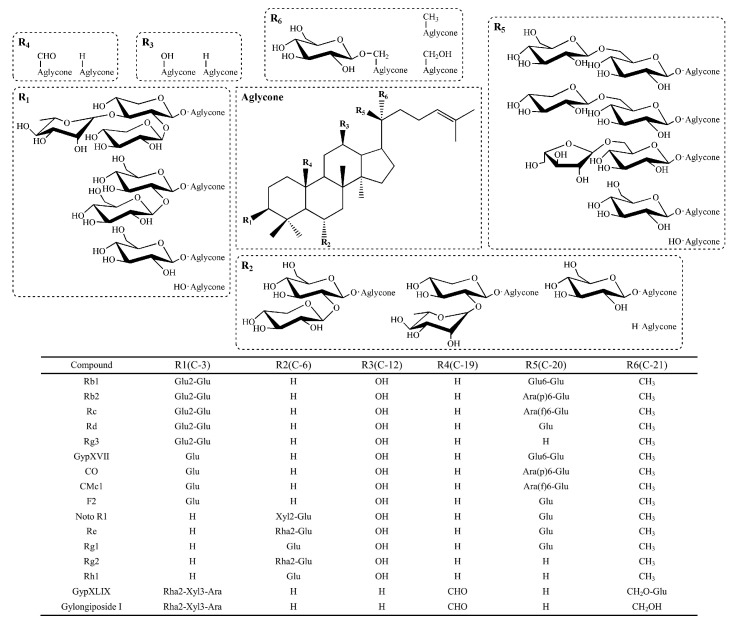
The chemical structures and structural information of ginsenosides and gypenosides.

Gypenosides, dammarane-type saponins, are known to be the principal bioactive constituents of *G. pentaphyllum*. Interestingly, although it has no botanical relationship with ginseng plants, the structures of gypenosides are closely similar to that of ginsenosides in *Panax ginseng*. As seen in Figure 9, ginsenosides and gypenosides all possess a dammarane-type triterpenoid as an aglycone. The structural difference mainly lies in the linked positions, numbers and types of sugar moieties in saponins. In order to better understand the biotransformation activity and substrate specificity of the recombinant cellulase from *F. pennivorans* DSM9078, ginsenosides including Rb1, Rb2, Rc and Rd were also used as substrates to test its hydrolytic activities. One unit (IU) of activity was defined as the amount of enzyme catalyzing the conversion of 1 μmol of ginsenoside substrate per min. Based on HPLC quantitative analysis, the recombinant cellulase could catalyze the hydrolysis of ginsenoside Rb1, Rb2, Rc and Rd. The order of the relative activity from high to low is gypenoside XLIX (378% ± 5.13) > Rd (100% ± 1.27) > Rb1 (68% ± 1.51) > Rb2 (27% ± 3.28) > Rc (25% ± 0.73). As the recombinant enzyme exhibited highest activity towards gypenoside XLIX, the large-scale transformation of gypenoside XLIX was chosen for further studied. Furthermore, the biotransformation activity of the recombinant enzyme towards ginsenoside Rb1, Rb2, Rc and Rd makes it possible to transform more lots of different types of saponins and enlarge its application scope.

There is currently no clinical drug against EV-A71 infection, and HFMD treatment is mostly restricted to antipyretic drugs, intravenous non-immune immunoglobulin, and glucocorticoids [28]. Medicinal plants have long been known for their protective effects against a broad range of bacteria, protozoa, parasites, and viruses [29]. We demonstrated the anti-viral activities of gypenoside XLIX and gylongiposide I against EV71, which lay a foundation for their usages as an alternative therapeutic compound in the treatment of EV71 infection. Compared with gypenoside XLIX, gylongiposide I exhibited enhanced inhibitory activity against EV71, indicating that deglycosylation of gypenoside results in its improved pharmaceutical activity. In our previous work, we also demonstrated that deglycosylated ginsenosides (Rd, GypXVII, and PPT) had significantly greater anti-inflammatory activity than their glycosylated precursors (Rb1, Re and Rg1) [30]. Hence, the biotransformation of glycosylated ginsenosides or gypenosides will be a promising method of generating deglycosylated compounds with new or improved pharmaceutical activities.

## 4. Materials and Methods

### 4.1. Materials

*Escherichia coli* strains were incubated at 37 °C in Luria-Bertani (LB) medium (10 g/L tryptone, 5 g/L yeast extract, and 10 g/L NaCl) with 50 mg/L kanamycin. Authentic gypenoside XLIX, ginsenoside Rb1, Rb2, Rc and Rd were purchased from Shanghai Yuanye Biological Technology Co. Ltd. (Shanghai, China).

The strain of EV71 CC077 was a gift from Professor Baisong Zheng of Jilin University, Changchun. The viruses were propagated in African green monkey kidney cells (Vero). Vero (No CCL81) and human rhabdomyosarcoma RD (No CCL136) cells were purchased from the ATCC (Manassas, VA, USA) and were maintained in Dulbecco’s modified Eagle’s medium (DMEM) (Hyclone, Logan, UT, USA) supplemented with 10% fetal bovine serum (FBS) (GIBCO BRL, Grand Island, NY, USA).

### 4.2. Molecular Cloning, Expression and Purification of the Cellulase from Fervidobaterium pennivorans DSM9078

Genomic DNA from *F. pennivorans* DSM9078 was extracted using a genomic DNA extraction kit (TIANGEN, Beijing, China) and used as a template of the gene cloning. The cellulase gene from *F. pennivorans* DSM9078 was amplified via polymerase chain reaction (PCR). The primers were designed based on genomic sequence (Genbank No. WP_245530410.1): upstream (5′-CAGCAGGGATCCATGGATCAGTCAGTTGCT-3′) and downstream (5′-CAGCAGCTCGAGTTATTCTTTGCTTTCTCCAA-3′) with *Bam*HI and *Xho*I restriction sites (underlined), respectively. The PCR amplified DNA fragment was purified and inserted into the pET28a vector digested with *Bam*HI and *Xho*I. The recombinant plasmid was transformed into *E. coli* BL21 (DE3), which was induced with 1 mM isopropyl β-D-thiogalactopyranoside (IPTG) at 16 °C for an additional 12 h.

The recombinant strains were collected by centrifugation at 6000× *g* for 20 min and resuspended in a lysis buffer (50 mM Tris-HCl, pH 7.1). The cells were then sonicated and centrifuged at 14,000× *g* for 30 min at 4 °C to remove the debris. The supernatants containing the target proteins were loaded onto a Ni-NTA affinity chromatography column (GE Healthcare) and purified using a 20–100 mM imidazole gradient. The purified enzyme was dialyzed against 50 mM phosphate–citrate buffer (pH 6.0) and concentrated to 1.0 mg/mL. The protein homogeneity was confirmed by 10% sodium dodecyl sulfate polyacrylamide gel electrophoresis (SDS-PAGE) (Figure S1).

### 4.3. Hydrolytic Activity of the Recombinant Enzyme

The cellulase activity of the recombinant enzyme was assayed according to the Ghose [31] and DNS [32] methods. The cellulase substrates include CMC (carboxymethyl cellulose sodium salt, medium viscosity; Fluka), and Avicel (PH-101; Fluka) were used to test the endoglucanase and exoglucanase activity of the recombinant enzyme, respectively. Protein concentrations were determined by using the Bradford Protein Assay Kit (Sangon Biotech, Shanghai, China). The reaction mixture composed of 1% CMC (*w/v*) or and 1 mg/mL enzyme in 50 mM phosphate–citrate buffer (pH 6.0). After incubation at 95 °C for 5 min, DNS was added to terminate the action, and the mixture was boiled in 100 °C water for 5 min. The absorption of the reaction mixture was measured at 540 nm. One unit (IU) of enzyme activity was defined as the amount of enzyme that released 1 μmol reducing sugars per min.

The *p*-nitrophenyl β-d-glucopyranoside (*p*NPG) was used to test the β-glucosidase activity of the enzyme. Hydrolysis of *p*NPG was measured at 95 °C in 50 mM phosphate–citrate buffer (pH 6.0). The activity was determined by measuring the increase in absorbance at 405 nm due to the release of *p*NP. One unit (IU) of activity was defined as the amount of enzyme liberating 1 μmol of *p*-nitrophenol per min.

The transformation activity of the recombinant enzyme towards gypenoside XLIX was studied. Enzyme solution (1 mg/mL) was reacted with equal volume of gypenoside XLIX (1 mg/mL) in 50 mM phosphate–citrate buffer (pH 6.0) at 95 °C for 5–30 min. The reaction solution without enzyme served as blank control. The activity was determined by measuring the substrate decrease via HPLC analysis. One unit (IU) of activity was defined as the amount of enzyme catalyzing the conversion of 1 μmol of gypenoside XLIX per min.

### 4.4. Characterization of the Recombinant Enzyme for Gypenoside XLIX Transformation

In order to determine the optimal condition for the biotransformation of gypenoside XLIX, the pH and temperature were optimized according to the method described previously. The optimal pH was tested over the pH range of 4.0–8.0 in 50 mM phosphate–citrate buffer at 60 °C with a stepwise of 0.5. Additionally, the optimal temperature was measured in the range 30–100 °C (5 °C intervals) at the optimum pH.

The pH stability of the enzyme was tested over the pH range of 4.0–8.0 in 50 mM phosphate–citrate buffer. The purified enzyme (1.0 mg/mL) was incubated in different buffers for 1 h. The residual activity on gypenoside XLIX was determined at 95 °C in 50 mM phosphate–citrate buffer (pH 6.0). Thermal stability of the enzyme was studied by incubating about 1.0 mg/mL purified enzyme solutions at different temperatures ranging from 30 °C to 100 °C for 12 h in 50 mM phosphate–citrate buffer (pH 6.0). The residual activity on gypenoside XLIX was determined at 95 °C in 50 mM phosphate–citrate buffer (pH 6.0).

### 4.5. Structural Analysis of the Biotransformed Product of Gypenoside XLIX

#### 4.5.1. HPLC Analysis

Chromatographic separation was performed using a Thermo Syncronis C_18_ column (150 mm × 2.1 mm, 1.9 μm) at 35 °C in an Agilent 1200 series HPLC system (Agilent Technologies, Santa Clara, CA, USA) at 35 °C. Water and acetonitrile were used as the mobile phases A and B, respectively. The gradient elution was programmed as follows, with a flow rate of 0.3 mL/min: 0–2.5 min, 19% (B); 2.5–5 min, 19–30% (B); 5–11 min, 30–33% (B); 11–20 min, 33–45% (B); 20–25 min, 45–65% (B). The injection volume was set at 3 µL.

#### 4.5.2. UPLC-QqQ-MS Analysis

UPLC-MS analysis was performed on a Thermo Ultimate 3000 system coupled with a TSQ Endura triple quadrupole mass spectrometer (Thermo Fisher Scientific, San Jose, CA, USA). The mobile phase of LC analysis consisted of formic acid/water (1:1000, *v/v*, A) and acetonitrile (B). The chromatographic column and separation conditions were the same as that used in the HPLC analysis.

Mass spectrometry analysis was performed using an electrospray ionization (ESI) source. Full-scan spectra were acquired from *m/z* 100 to 2000 in the negative ion mode. The spray voltage and vaporizer temperature were set to 2500 V and 317 °C, respectively. The flow rates of sheath gas, auxiliary gas and sweep gas were set to 40, 11, and 1 arbitrary units, respectively. Tandem mass spectrometry was performed using argon as the collision gas at collision energy of 40 eV. All the tandem mass spectra were recorded from *m/z* 200 to 1100. MS Data were analyzed using Thermo Xcalibur software, version 2.2 SP1.48 (Thermo Fisher Scientific Inc., San Jose, CA, USA).

#### 4.5.3. ^13^C NMR Analysis

Before being subjected to NMR analysis, the biotransformed product was isolated to a purity of over 95% (HPLC) using a Waters prep-HPLC system (Waters, Milford MA, USA) with a Waters Sunfire Prep C18 OBD^TM^ column (19 mm × 50 mm, 5 μm). Isocratic elution was performed at room temperature using a mixture of ACN/H_2_O (46:54, *v/v*) at a flow rate of 15 mL/min, with UV detection at 203 nm. The injection volume was set at 200 µL. ^13^C NMR spectra were recorded on a Bruker AVIII 600 MHz spectrometer at an operating frequency of 151 MHz (Bruker BioSpin, Bremen, Germany).

### 4.6. Large-Scale Production of Gylongiposide I by Using the Recombinant Enzyme

High-cell-density fermentation was performed to produce the recombinant enzyme. The LB medium supplemented with kanamycin (50 mg/mL) was used to cultivate the recombinant *E. coli* BL21 (DE3) cells in a 5 L tank reactor (FS-05-D05D05P, Winpact, CA, USA) with a 2 L working volume. At the initial stage, fermentation was performed at 37 °C, pH 7.0 and at stirring speed of 200 rpm. When dissolved oxygen was lower than 30%, the speed increased by 10 rpm. When the speed reached 900 rpm, the dissolved oxygen coupling was closed. After the nutrition was exhausted, the dissolved oxygen feedback feeding was carried out by flow feeding to ensure the dissolved oxygen range remained at 20−40%. When OD600 reached 30, Cells were induced at 25 °C for 12 h and were then harvested via centrifugation at 5000 rpm for 20 min at 4 °C. Cell pellets were resuspended in 10 volumes (*w/v*) of 50 mM phosphate–citrate buffer (pH 6.0) and disrupted by sonication, and the supernatant was used as the crude enzyme for biotransformation reaction.

In order to optimize the enzyme and substrate concentration, enzyme concentrations from 2.5 to 20 mg/mL (at 25 mg/mL gypenoside XLIX) and substrate concentrations from 0 to 60 mg/mL (at 20 mg/mL enzyme) were evaluated. The time course reactions of gylongiposide I production were performed at 80 °C in 50 mM phosphate–citrate buffer (pH 6.0) containing 35 mg/mL gypenoside XLIX and 20 mg/mL enzyme.

The scaled-up biotransformation of gypenoside XLIX into gylongiposide I was performed in a 5.0 L glass bottle (2.0 L working volume) under optimal conditions (shaking 200 rpm for 4 h at pH 6.0 and 80 °C). The crude recombinant enzyme (20 mg/mL) was reacted with an equal volume of gypenoside XLIX (35 mg/mL). Samples were collected at regular intervals and HPLC was used to monitor the biotransformation process.

### 4.7. Separation and Purification of the Transformed Gylongiposide I

After the scaled-up biotransformation of gypenoside XLIX, equal volume of 95% ethanol was added in the glass bottle to end the enzymatic reaction. The mixture was cooled at 4 °C and centrifuged at 5000 rpm for 20 min. The supernatant was dried by rotary evaporation and weighted.

HP-20 macroporous resin fillings were used for the separation of gylongiposide I transformed from gypenoside XLIX by using the recombinant enzyme. The reaction mixture was redissolved in water and poured slowly into the column in order to remove the enzymes, salts, and free sugars. After static adsorption for 1 h, elution was conducted using water (10 times of the column volume) and 70% ethanol (*v/v*, 8 times of the column volume) successively with a flow rate of 10 mL/min. The chromatographic fractions of 70% ethanol were collected and evaporated to dryness for further purification using 200–300 mesh silica gel column. The sample and the silica gel was mixed with a mass ratio of 1:20, and then eluted using chloroform:methanol:water = 22:8:1 (lower phase) as solvent. The eluent was collected and dried by lyophilization for activity experiment.

### 4.8. Cytotoxicity Assays

RD cells in 96-well plates were cultured to 80 to 90% confluence (approximately 5 × 104 cells/well). Then, the cells were treated with different concentrations of gypenoside XLIX and gylongiposide I dissolved in dimethyl sulfoxide for 48 h. Cell Counting kit-8 (CCK-8; Beyotime, Shanghai, China) was used to measure the cell viability according to product description. The absorbance at 450 nm wavelength was measured using a microplate reader. The percentage of cell viability was calculated as follows: percentage of cell viability = (Atreatment-Ablank)/(Acontrol-Ablank) × 100% (where, A = absorbance).

### 4.9. Antiviral Activity In Vitro

RD cells plated into 24-well culture plates were cultured for 12 h. Then, the medium was removed, and cells were infected with the EV71 (multiplicity of infection [MOI], 2) for 4 h. Cells were washed three times with DMEM to removed unabsorbed virus and then treated with different concentrations of gypenoside XLIX and gylongiposide I. After 48 h, virus-infected cells and culture supernatants were collected for Western blotting, qRT-PCR, and virus titer detection.

### 4.10. Western Blot Analysis

Samples of virus-infected RD cells or cultural supernatant were treated with 1×loading buffer (0.08 M Tris [pH 6.8] with 2.0% SDS, 10% glycerol, 0.1 M dithiothreitol, and 0.2% bromophenol blue) and boiled at 100 °C for 10 min. After centrifugation, samples were further subjected to SDS-PAGE. Then, proteins in the SDS-PAGE gel was transferred to polyvinylidene fluoride membrane (catalog no. BSP0161; Pall) and detected with corresponding primary and alkaline phosphatase-conjugated secondary antibody. The membranes were then reacted with 5-bromo-4-chloro-39-indolylphosphate (BCIP) and nitro-blue tetrazolium (NBT) substrate (Sigma-Aldrich, St. Louis, MO, USA). Polyclonal antibody against EV71 was obtained from rabbits immunized with EV71 CC077. Anti-tubulin monoclonal antibody was purchased from Abcam (MAb; ab11323, Cambridge, UK). Mouse and rabbit secondary antibodies were obtained from Proteintech.

### 4.11. Quantitative Real-Time RT-PCR

RNA was extracted from infected cells using Trizol reagent (Gibco-BRL, Rockville, MD, USA). Then, 200 ng of total RNA was reverse-transcribed with oligo dT primers using the High Capacity cDNA RT Kit (Applied Biosystems) in a 20 μL cDNA reaction. For quantitative PCR, the template cDNA was added to a 20 μL reaction with SYBR GREEN PCR Master Mix (Applied Biosystems) and 0.2 mM of primer. The amplification was carried out using an ABI Prism 7000 for 40 cycles under the following conditions: an initial denaturation of 95 °C for 10 min, plus 40 cycles of 95 °C for 15 s, then 60 °C for 1 min. The fold changes were calculated relative to GAPDH using the ∆∆Ct method for EV71 VP1 mRNA analysis.

### 4.12. Plaque Assay

RD cells (1 × 10^5^ cells/well) were seeded in 24-well plates, incubated at 37 °C for 16–18 h, and 10-fold serial dilution of viral sample was added to each well. After absorption for 1 h at 37 °C, overlay medium containing 2% FBS and 0.8% methylcellulose were added and incubated at 37 °C for 72 h. The overlay medium was discarded and stained with a solution containing 4% formaldehyde and 1% crystal violet in PBS at room temperature for 1 h. The plates were washed with flowing water and dried to count plaques. Plaque-forming unit per milliliter (PFU/mL) was used to calculate viral titers which were multiplied by dilution factors.

### 4.13. Statistical Analysis

Statistical analyses were conducted using SPSS 17.0 software. Data are reported as the means ± SDs from three independent experiments. Statistical significance was evaluated by Student’s *t*-test. Significant differences are indicated in figures as follows: * *p* ≤ 0.05, ** *p* ≤ 0.01, ns stands for not significant.

## 5. Conclusions

In the current study, a thermophilic cellulase was successfully cloned and expressed in *Escherichia coli*. The gram-scale production of gylongiposide I from gypenoside XLIX was achieved by using the recombinant enzyme with a molar yield of 100%. Moreover, gylongiposide I and gypenoside XLIX were proven to be novel potent anti-EV71 candidates. In the future, the ideal transformation properties of the recombinant cellulase will make it a promising tool to produce more valuable deglycosylated saponins.

## Data Availability

Data is contained within the article or Supplementary Materials.

## Supplementary Materials

The following supporting information can be downloaded at: https://www.mdpi.com/article/10.3390/molecules27134094/s1, Table S1. ^13^C NMR dates of the product transformed from gypenoside XLIX by using the recombinant enzyme; Figure S1: Purification of the recombinant protein. Lane M, molecular mass marker; Lane 1, purified protein after Ni-NTA affinity chromatography purification.

## Sample Availability

Samples of the compounds gypenoside XLIX and gylongiposide I are available from the authors.
