# Supplementary material for: Enzymatic Biotransformation of Gypenoside XLIX into Gylongiposide I and Their Antiviral Roles against Enterovirus 71 In Vitro
Source: Molecules. 2022 Jun 25;27(13):4094. doi: 10.3390/molecules27134094 (PMC9268165; doi:10.3390/molecules27134094)
Supplement: Supplementary file 1 [file molecules-27-04094-s001.zip › molecules-1768679-supplementary.pdf]

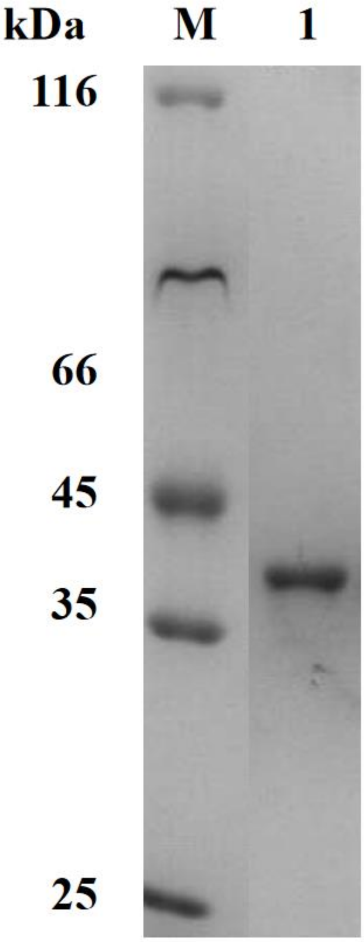

Figure S1 Purification of the recombinant protein. Lane M, molecular mass marker; Lane 1, purified protein after Ni-NTA affinity chromatography purification.

Table S1. ^{13}C NMR dates of the product transformed from gypenoside XLIX by using the recombinant enzyme (151 MHz, MeOD, *δ*, ppm).

**Positon**	***δ*C, type**	**Positon**	***δ*C, type**	**Positon**	***δ*C, type**
1	33.0, CH_2	17	45.0, CH	Ara-3	76.3, HC-O
2	26.7, CH_2	18	14.9, CH_3	Ara-4	65.4, CH
3	87.0, CH	19	206.6, C=O	Ara-5	54.6, CH_2
4	40.0, C	20	73.9, C	Rha-1	100.6, O-C-O
5	52.6, CH	21	63.0, CH_2	Rha-2	70.7, CH
6	16.4, CH_2	22	35.1, CH_2	Rha-3	69.6, CH
7	34.0, CH_2	23	22.0, CH_2	Rha-4	70.7, CH
8	39.5, C	24	124.6, C=C	Rha-5	67.6, CH
9	52.5, CH	25	130.6, C=C	Rha-6	17.0, CH_3
10	49.7, C	26	24.5, CH_3	Xyl-1	103.8, O-C-O
11	21.6, CH_2	27	16.6, CH_3	Xyl-2	73.2, CH
12	23.6, CH_2	28	25.5, CH_3	Xyl-3	76.1, CH
13	40.9, CH	29	15.4, CH_3	Xyl-4	68.9, CH
14	48.2, C	30	16.1, CH_3	Xyl-5	67.6, CH_2
15	31.3, CH_2	Ara-1	103.7, O-C-O		
16	27.1, CH_2	Ara-2	72.3, HC-O		
